# Substance use disorders among refugee and non-refugee immigrants in Sweden

**DOI:** 10.1093/eurpub/ckag152

**Published:** 2026-08-17

**Authors:** Kenta Okuyama, Sara Larsson Lönn, Ardavan M Khoshnood, Shervin Assari, Jan Sundquist, Kristina Sundquist

**Affiliations:** Department of Clinical Sciences Malmö, Center for Primary Health Care Research, Lund University, Malmö, Sweden; University Clinic Primary Care, Skåne University Hospital, Region Skåne, Sweden; Department of Clinical Sciences Malmö, Center for Primary Health Care Research, Lund University, Malmö, Sweden; University Clinic Primary Care, Skåne University Hospital, Region Skåne, Sweden; Department of Clinical Sciences Malmö, Center for Primary Health Care Research, Lund University, Malmö, Sweden; University Clinic Primary Care, Skåne University Hospital, Region Skåne, Sweden; Department of Clinical Sciences Malmö, Emergency Medicine, Lund University, Skåne University Hospital, Malmö, Sweden; Department of Internal Medicine, Charles R. Drew University of Medicine and Science, Los Angeles, CA, United States; Department of Family Medicine, Charles R. Drew University of Medicine and Science, Los Angeles, CA, United States; Department of Urban Public Health, Charles R. Drew University of Medicine and Science, Los Angeles, CA, United States; Marginalization Related Diminished Returns Research Center, Los Angeles, CA, United States; Department of Clinical Sciences Malmö, Center for Primary Health Care Research, Lund University, Malmö, Sweden; University Clinic Primary Care, Skåne University Hospital, Region Skåne, Sweden; Department of Clinical Sciences Malmö, Center for Primary Health Care Research, Lund University, Malmö, Sweden; University Clinic Primary Care, Skåne University Hospital, Region Skåne, Sweden

## Abstract

Evidence is inconclusive on whether immigrants are at higher risk of substance use disorders compared to non-immigrants. We investigated the risk of drug and alcohol use disorder (DUD and AUD) in first- and second-generation immigrants with refugee and non-refugee backgrounds using the nationwide Swedish longitudinal healthcare and legal registers. We included 798 141 males and 756 903 females who turned 16 years of age between 2005 and 2020. DUD and AUD were identified between ages 16 and 25 years using healthcare and legal registers. Immigrants were categorized into first- and second-generation refugees and non-refugees. Cox proportional hazard models estimated DUD and AUD risk in relation to immigrant status (non-immigrants as reference), adjusting for covariates. Sensitivity analyses were conducted using healthcare register data only. DUD risks were higher among most refugee and non-refugee immigrants compared to non-immigrants, i.e. hazard ratios (HRs) ranged from HR: 1.05, 95% CI: 0.95–1.15 (first-generation female refugees) to HR: 2.63, 95% CI: 2.54–2.73 (first-generation male refugees). AUD risks among immigrants were lower or similar to non-immigrants, i.e. HRs ranged from HR: 0.55, 95% CI: 0.49–0.62 (first-generation female refugees) to HR: 1.17, 95% CI: 1.03–1.34 (first-generation male non-refugees). Sensitivity analyses restricted to healthcare registers yielded somewhat attenuated associations for DUD, particularly among first-generation male refugees. Refugee and non-refugee immigrants in Sweden were generally at elevated risk of DUD, whereas AUD risks were largely similar to or lower than that of non-immigrants. The findings were broadly robust in analyses restricted to healthcare data.

## Introduction

People migrate for different reasons, e.g. to pursue education, work opportunities, reunite with family, and flee from distressing circumstances such as armed conflicts, political persecution, and natural disasters. Currently, 281 million people live in a country other than their country of birth, and among those, 35.4 million people are refugees [1]. Both refugee and non-refugee immigrants are considered to have an increased vulnerability to develop impaired psychiatric health due to various stress factors before, during, and after their migration [2]. Systematic reviews and meta-analyses have indicated a higher risk of depression, anxiety, and psychotic disorders among refugee and non-refugee immigrants compared to non-immigrants [3–5].

An impaired psychiatric health could sometimes be related to substance use disorder that is increasing globally [6]. In Europe, it is especially prevalent among the youngest population groups [7]. While migration-related stressors, trauma, and post-migration adversities may increase vulnerability to substance use and disorders among some immigrant groups [8, 9], several studies have reported a lower risk of substance use and substance use disorders among refugee and non-refugee immigrants [10–12]. These paradoxical findings could be explained by the so-called healthy immigrant effect, i.e. those who migrate are highly motivated and resilient and therefore, healthier than non-immigrants [13]. Nevertheless, methodological limitations in previous studies may have hampered the validity of these findings. For example, many studies have been cross-sectional, performed on small samples of the population, not distinguishing between refugees and non-refugees, and relying on self-reported data. One study overcame these shortcomings by using nationwide longitudinal population and medical registers, and the findings indicated a lower risk of drug use disorders (DUD) and alcohol use disorders (AUD) among refugee and non-refugee immigrants [11]. The study measured DUD and AUD by medical diagnoses only and that could have underestimated the risk among immigrants. Immigrants may be less likely to seek healthcare services, partly due to language and cultural barriers, as well as due to fear of stigmatization and deportation [14]. In order to mitigate the underestimation of the risk of substance use disorders, using multiple types of register data, e.g. healthcare and legal, could be an advantage [15]. In the present study, we focused on substance use disorders rather than substance use in general. Substance use disorders were primarily identified through healthcare records, but we also incorporated legal register data that capture substance-related events as additional indicators of substance use disorders. This broader register-based approach aimed to improve case ascertainment while acknowledging that legal registrations are not equivalent to a clinical diagnosis.

Given this background, we aimed to investigate whether young refugee and non-refugee immigrants are at a higher risk of DUD and AUD by using multiple types of nationwide longitudinal register data.

## Methods

We used multiple nationwide longitudinal Swedish registers linked by pseudonymized serial numbers that ensured the integrity of the included individuals. Our study followed the STROBE guidelines for observational studies. The study was approved by the Swedish Ethical Review Authority (no. 2021-04268).

### Data sources

The registers used in this study include the National Patient Register (Inpatient and Outpatient registers), the Prescribed Drug Register, the Crime Register, the Suspicion Register, the Multi-Generation Register, the Longitudinal Integration Database for Health Insurance and Labour Market Studies (LISA), the Total Population Register, and the Migration register (containing information on country of birth and reason for migration). These registers provided information on healthcare diagnoses, prescribed medications, criminal convictions and suspicions, family relationships, demographic characteristics, educational attainment, and migration-related factors. Detailed information on each register can be found in the Supplementary  Table S1.

### Study individuals

**Figure 1:**
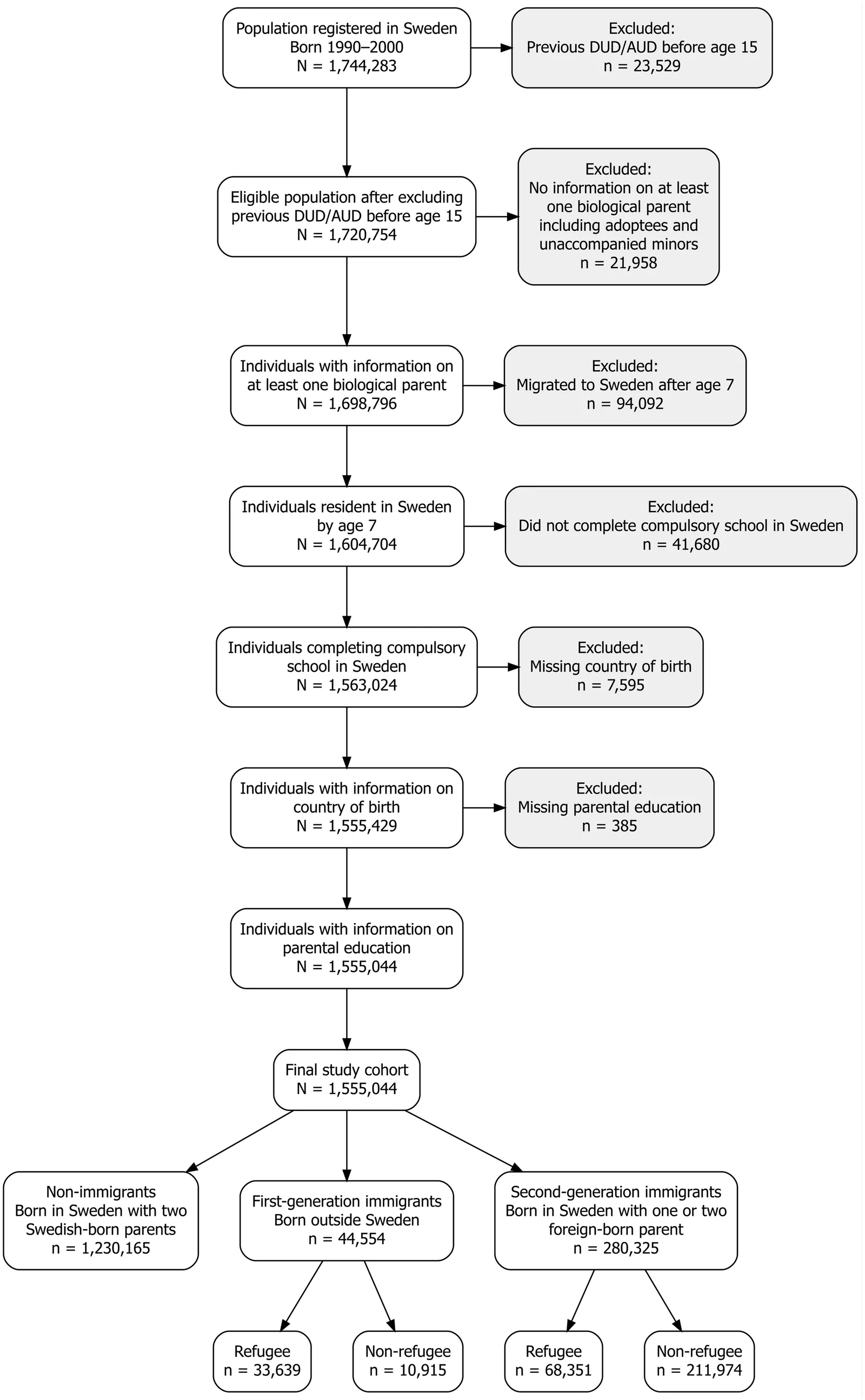
Flow chart of study individuals.

We included individuals who were born between 1990 and 2000, without previous DUD and AUD at the age of 15. They were followed from the age of 16 until the first identification of DUD, AUD, death, emigration, age 25, or the end of the study period (2020), whichever came first (Supplementary Fig. S1). Individuals who migrated to Sweden after age 7 years, did not complete compulsory school, unaccompanied minors, adoptees, and individuals with missing information on country of birth or parental education were excluded. Individuals who did not complete compulsory school in Sweden were also excluded because the present study was based on an existing nationwide cohort that was defined using this eligibility criterion. This criterion was therefore retained to preserve the same predefined study population. The remaining exclusions were made because immigrant status and parental characteristics could not be reliably classified from the available register data (Fig. 1).

### Outcome

DUD and AUD were assessed by Inpatient, Outpatient, Crime, Suspicion, and Prescribed Drug registers which were available between 2005 and 2020. They were identified by WHO’s International Classification of Diseases (ICD) codes from the Inpatient and Outpatient registers, crime and suspicion codes from the Crime and Suspicion registers, as well as the Anatomical Therapeutic Chemical codes from the Prescribed Drug register. These are aligned with previous studies [16, 17], and detailed information of the register data can be found in the Supplementary  Table S2. Healthcare registers captured clinically diagnosed substance use disorders, whereas legal registers captured substance-related events that were used as additional indicators of substance use disorders. This broader register-based approach has previously been used in Swedish register studies to improve case ascertainment but the present study also included the Suspicion Register [15]. The Swedish National Patient Register has generally been shown to have good diagnostic validity across a wide range of conditions, although validity varies between diagnoses [18, 19]. To assess the potential influence of using legal registers for outcome ascertainment, we conducted a sensitivity analysis in which DUD and AUD were identified using healthcare registers only.

### Exposure

From the registers, immigrant status was assessed based on the country of birth of individuals or their parents as well as their reasons for migration. The study individuals were categorized as follows: Non-immigrants—those who were born in Sweden with two Swedish-born parents; First-generation immigrants—those who were born outside of Sweden; and Second-generation immigrants—those who were born in Sweden with one or two foreign-born parents. First- and second-generation immigrants were further categorized as refugee or non-refugee immigrants based on their parents’ reasons for migration that were registered according to the UN Convention Relating to the Status of Refugees, Swedish legislation, and EU regulations [20]. Specifically, they were categorized as refugee immigrants if one of their parents’ reasons for migration was either need for protection, humanitarian reasons, or other refugees, and as non-refugee immigrants if both of their parents’ reasons for migration was either work, studies, or family unification. In supplementary analysis, region of origin was taken into account, i.e. six regions based on the country of birth [21].

### Covariates

Parental substance use disorder and parental education were included in the analyses. These covariates were identified as potential confounders for the association between immigrant status and DUD and AUD as illustrated in a directed acyclic graph (Supplementary Fig. S2) [22]. Parental substance use disorder was defined as either DUD or AUD and defined as above. Parental education level was assessed based on the highest education attained by any of the parents (Low: compulsory school, Middle: high school, High: university or more). In addition, we included birth year as a covariate to account for potential differences in social and environmental conditions across birth cohorts.

### Statistical analysis

Cox proportional hazards models were applied to estimate the risk of DUD and AUD with time to the first identification of DUD or AUD as an outcome. Each group of immigrants was compared to non-immigrants in separate analyses. Parental substance use disorder, parental education, and birth year were included as covariates in the adjusted models. Analyses were stratified by the individuals’ sex as the incidence of DUD and AUD is known to differ by sex [23]. The proportional hazard assumption was assessed using Schoenfeld Residuals, and no evidence of violation was observed.

## Results

Our study individuals consisted of 798 141 males and 756 903 females who became 16 years old between 2005 and 2020. Among them, 20.79% of males and 21.0% of females were first- or second-generation immigrants.

Table 1 presents the characteristics of the study individuals. The proportion of low parental education was higher among refugee and non-refugee immigrants than among non-immigrants. Refugee immigrants’ regions of origin were predominantly Middle East or North Africa followed by Eastern Europe. Non-refugee immigrants were mostly from Western countries followed by Eastern Europe and Asia or Oceania.

**Table 1:** Characteristics of study individuals by immigrant status.

	Non-immigrants	First-generation		Second-generation	
	Native	Refugee	Non-refugee	Refugee	Non-refugee
Males					
* N* (%)	632 240 (79.21%)	17 314 (2.17%)	5521 (0.69%)	34 560 (4.33%)	108 506 (13.59%)
Parental substance use disorder, *n* (%)	90 791 (14.36%)	1383 (7.99%)	476 (8.62%)	4478 (12.96%)	20 747 (19.12%)
Parental education, *n* (%)					
Low	15 697 (2.48%)	2866 (16.55%)	776 (14.06%)	3732 (10.80%)	7104 (6.55%)
Middle	172 904 (27.35%)	3901 (22.53%)	1077 (19.51%)	8511 (24.63%)	29 646 (27.32%)
High	443 639 (70.17%)	10 547 (60.92%)	3668 (66.44%)	22 317 (64.57%)	71 756 (66.13%)
Regions of origin, *n* (%)					
Africa	–	1282 (7.40%)	333 (6.03%)	4138 (11.97%)	3556 (3.28%)
Asia or Oceania	–	1454 (8.40%)	1118 (20.25%)	3329 (9.63%)	16419 (15.13%)
Eastern Europe	–	6650 (38.41%)	1419 (25.70%)	8807 (25.48%)	15361 (14.16%)
Latin America or the Caribbean	–	649 (3.75%)	369 (6.68%)	3214 (9.30%)	5670 (5.23%)
Middle East or North Africa	–	6805 (39.30%)	369 (6.68%)	14 944 (43.24%)	10 113 (9.32%)
Western	–	474 (2.74%)	1913 (34.65%)	128 (0.37%)	57 387 (52.89%)
Females					
* N* (%)	597 925 (79.00%)	16 325 (2.16%)	5394 (0.71%)	33 791 (4.46%)	103 468 (13.67%)
Parental substance use disorder, *n* (%)	87 208 (14.59%)	1273 (7.8%)	508 (9.42%)	4274 (12.65%)	19 992 (19.32%)
Parental education, *n* (%)					
Low	15 324 (2.56%)	2739 (16.78%)	769 (14.26%)	3696 (10.94%)	6826 (6.60%)
Middle	164 674 (27.54%)	3568 (21.86%)	1070 (19.84%)	8302 (24.57%)	28 446 (27.49%)
High	417 927 (69.9%)	10 018 (61.37%)	3555 (65.91%)	21 793 (64.49%)	68 196 (65.91%)
Regions of origin, *n* (%)					
Africa	–	1201 (7.36%)	339 (6.28%)	4094 (12.12%)	3638 (3.52%)
Asia or Oceania	–	1434 (8.78%)	1189 (22.04%)	3334 (9.87%)	15 260 (14.75%)
Eastern Europe	–	6184 (37.88%)	1359 (25.19%)	8479 (25.09%)	14 861 (14.36%)
Latin America or the Caribbean	–	587 (3.60%)	322 (5.97%)	3129 (9.26%)	5349 (5.17%)
Middle East or North Africa	–	6486 (39.73%)	337 (6.25%)	14 614 (43.25%)	9663 (9.34%)
Western	–	433 (2.65%)	1848 (34.26%)	141 (0.42%)	54 697 (52.86%)

### Risk of DUD and AUD by immigrant status

Table 2 presents the crude and adjusted hazard ratio (HR) with 95% confidence interval (CI) for DUD and AUD by immigrant status. In the adjusted models among males, first- and second-generation refugee immigrants had higher hazards of DUD than non-immigrants, i.e. HR: 2.63 (95% CI: 2.54–2.73) and HR: 1.97 (95% CI: 1.94–2.01), respectively. First- and second-generation male non-refugee immigrants had also higher hazards of DUD than non-immigrants, i.e. HR: 2.12 (95% CI: 1.98–2.27) and HR: 1.37 (95% CI: 1.33–1.41), respectively. Among females, second-generation refugee immigrants had higher hazards of DUD than non-immigrants, i.e. HR: 1.38 (95% CI: 1.33–1.42). First- and second-generation female non-refugee immigrants had also higher hazards of DUD than non-immigrants, i.e. HR: 1.65 (95% CI: 1.45–1.87) and HR: 1.32 (95% CI: 1.25–1.40), respectively. Hazards of DUD among first-generation female refugee immigrants were not different from non-immigrants. For AUD among males, first- and second-generation non-refugee immigrants had slightly higher hazards of AUD than non-immigrants, i.e. HR: 1.17 (95% CI: 1.03–1.34), and HR: 1.13 (95% CI: 1.07–1.18), respectively. The hazards of AUD among first-generation male refugee immigrants were lower than in non-immigrants, i.e. HR: 0.88 (95% CI: 0.81–0.96), and the hazards of AUD among second-generation male refugee immigrants were not different from non-immigrants. Among females, first- and second-generation refugee immigrants had lower hazards of AUD than non-immigrants, i.e. HR: 0.55 (95% CI: 0.49–0.62) and HR: 0.94 (95% CI: 0.90–0.97), respectively. Second-generation female non-refugee immigrants had slightly higher hazards of AUD than non-immigrants, i.e. HR: 1.15 (95% CI: 1.09–1.22). The hazards of AUD among first-generation female non-refugee immigrants were not different from non-immigrants.

**Table 2:** Adjusted hazard ratio (HR) and 95% confidence interval (CI) for the first case of DUD and AUD between 16 and 25 years old by immigrant status.

			Crude model	Adjusted model^b^
		Number of cases (%)^a^	HR (95% CI)	HR (95% CI)
Males				
DUD				
Non-immigrants	Native	51 471 (8.14%)	Reference	Reference
First-generation	Refugee	3219 (18.59%)	2.43 (2.34–2.52)	2.63 (2.54–2.73)
	Non-refugee	859 (15.56%)	2.18 (2.04–2.33)	2.12 (1.98–2.27)
Second-generation	Refugee	6808 (19.70%)	2.17 (2.13–2.21)	1.97 (1.94–2.01)
	Non-refugee	14 963 (13.79%)	1.45 (1.41–1.50)	1.37 (1.33–1.41)
AUD				
Non-immigrants	Native	24 395 (3.86%)	Reference	Reference
First-generation	Refugee	575 (3.32%)	0.86 (0.79–0.93)	0.88 (0.81–0.96)
	Non-refugee	216 (3.91%)	1.12 (0.98–1.28)	1.17 (1.03–1.34)
Second-generation	Refugee	1108 (3.21%)	1.08 (1.05–1.11)	1.03 (1.00–1.07)
	Non-refugee	4874 (4.49%)	1.18 (1.12–1.24)	1.13 (1.07–1.18)
Females				
DUD				
Non-immigrants	Native	18 040 (3.02%)	Reference	Reference
First-generation	Refugee	454 (2.78%)	0.93 (0.84–1.02)	1.05 (0.95–1.15)
	Non-refugee	244 (4.52%)	1.66 (1.46–1.88)	1.65 (1.45–1.87)
Second-generation	Refugee	1373 (4.06%)	1.52 (1.48–1.57)	1.38 (1.33–1.42)
	Non-refugee	4692 (4.53%)	1.43 (1.35–1.51)	1.32 (1.25–1.40)
AUD				
Non-immigrants	Native	20 788 (3.48%)	Reference	Reference
First-generation	Refugee	295 (1.81%)	0.52 (0.46–0.58)	0.55 (0.49–0.62)
	Non-refugee	174 (3.23%)	1.01 (0.87–1.17)	1.07 (0.92–1.24)
Second-generation	Refugee	715 (2.12%)	0.96 (0.93–1.00)	0.94 (0.90–0.97)
	Non-refugee	4087 (3.95%)	1.20 (1.14–1.27)	1.15 (1.09–1.22)

a Proportion of cases to the number of individuals.

b Adjusted for birth year, parental substance use disorder, and parental education. Analyses were separately conducted for DUD and AUD, as well as males and females.

### Sensitivity analysis using healthcare data only

The sensitivity analysis based on healthcare data only yielded findings that were broadly consistent with the main analyses (Table 3), although the hazard ratios for DUD were somewhat attenuated. The attenuation was most pronounced among first-generation refugee male immigrants, for whom the associations were no longer statistically significant. Nevertheless, elevated DUD risk remained among other immigrant groups, particularly in second-generation immigrants. The patterns of AUD risks were largely unchanged, with most immigrant groups showing lower or similar risks compared with non-immigrants.

**Table 3:** Crude and adjusted hazard ratios (HR) and 95% confidence interval (CI) for the first case of DUD and AUD identified in healthcare registers only by immigrant status.

			Crude model	Adjusted model^b^
		Number of cases (%)^a^	HR (95% CI)	HR (95% CI)
Males				
DUD				
Non-immigrants	Native	7552 (1.19%)	Reference	Reference
First-generation	Refugee	181 (1.05%)	0.94 (0.81–1.08)	1.09 (0.94–1.26)
	Non-refugee	87 (1.58%)	1.54 (1.25–1.91)	1.53 (1.24–1.89)
Second-generation	Refugee	1691 (1.58%)	1.43 (1.36–1.51)	1.32 (1.25–1.39)
	Non-refugee	639 (1.77%)	1.53 (1.41–1.66)	1.43 (1.32–1.55)
AUD				
Non-immigrants	Native	20 430 (3.23%)	Reference	Reference
First-generation	Refugee	369 (2.13%)	0.66 (0.59–0.73)	0.69 (0.62–0.76)
	Non-refugee	166 (3.01%)	1.01 (0.87–1.18)	1.07 (0.91–1.24)
Second-generation	Refugee	3419 (3.19%)	1.01 (0.97–1.05)	0.98 (0.94–1.01)
	Non-refugee	1362 (3.78%)	1.18 (1.12–1.25)	1.13 (1.07–1.20)
Females				
DUD				
Non-immigrants	Native	7278 (1.22%)	Reference	Reference
First-generation	Refugee	159 (0.97%)	0.80 (0.69–0.94)	0.92 (0.79–1.08)
	Non-refugee	92 (1.71%)	1.57 (1.28–1.93)	1.57 (1.28–1.93)
Second-generation	Refugee	1706 (1.65%)	1.40 (1.33–1.47)	1.28 (1.22–1.35)
	Non-refugee	577 (1.71%)	1.43 (1.31–1.55)	1.33 (1.22–1.45)
AUD				
Non-immigrants	Native	19 674 (3.29%)	Reference	Reference
First-generation	Refugee	284 (1.74%)	0.53 (0.47–0.59)	0.56 (0.50–0.63)
	Non-refugee	165 (3.06%)	1.01 (0.86–1.17)	1.06 (0.91–1.24)
Second-generation	Refugee	3233 (3.12%)	0.97 (0.93–1.00)	0.94 (0.91–0.98)
	Non-refugee	1333 (3.95%)	1.21 (1.15–1.28)	1.16 (1.10–1.23)

a Proportion of cases to the number of individuals.

b Adjusted for birth year, parental substance use disorder, and parental education. Analyses were separately conducted for DUD and AUD, as well as males and females.

### Risk of DUD and AUD by immigrants’ regions of origin

When immigrants’ regions of origin were accounted for in a supplementary analysis, immigrants from certain regions were at a particularly higher risk of DUD and AUD (Supplementary Figs. S3 and S4). Among males, all first- and second-generation immigrant groups from Africa, Latin America or the Caribbean, and Middle East or North Africa were at 2× to 4× higher hazards of DUD than non-immigrants. For AUD, the magnitudes of the HRs for the different regions of origin were modest. Among females, immigrant groups from Africa, Latin America or the Caribbean, and Middle East or North Africa had higher hazards of DUD than non-immigrants. As for AUD in females, most female immigrants were at lower or similar hazards as non-immigrants, and the magnitudes of the HRs were modest.

## Discussion

Our findings indicate that among the young population aged 16–25 years, refugee and non-refugee immigrants were at a higher risk of DUD than non-immigrants, especially among male refugee immigrants. AUD risk among refugee and non-refugee immigrants was mostly not different or lower compared to non-immigrants.

Immigrants may be at risk of using drugs and alcohol as a coping strategy to deal with stress that can be ascribed to traumatic experiences, discrimination, and socioeconomic disadvantages [8]. In general, refugee immigrants are typically more exposed to these stress factors. Our findings showed that male refugee immigrants were at almost 2× higher hazards of DUD than non-immigrants. The findings were in line with previous studies that have reported a higher risk of developing other psychiatric disorders, e.g. schizophrenia and psychotic disorders, among refugees than non-refugee immigrants or non-immigrants [3, 9]. Unlike previous studies on substance use disorder, we distinguished refugee and non-refugee immigrants according to the officially registered reasons for migration and assessed DUD and AUD using information from both healthcare and legal registers. This broader register-based approach may improve case ascertainment because substance use disorders are not always identified through healthcare services alone [15]. However, legal register indicators may also be influenced by differences in criminal justice contact and surveillance.

To examine the potential influence of using legal register data on our findings, we conducted a sensitivity analysis using healthcare registers only. Although the estimated hazards were somewhat attenuated, the overall pattern of elevated DUD risk among most immigrant groups remained unchanged. The attenuation was most pronounced among first-generation refugee male immigrants, for whom the associations were no longer statistically significant when DUD was identified using healthcare register data only. One possible explanation is that first-generation refugees may be less likely to access healthcare services for substance-related problems because of language and cultural barriers, limited familiarity with the healthcare system, stigma, or other barriers to healthcare access [9]. Previous studies have reported lower mental healthcare utilization among refugees and asylum seekers in Europe despite substantial mental health needs [9, 14]. Consequently, healthcare registers alone may underestimate substance use disorders in this group. However, it is also possible that differences in the contact with the criminal justice system or surveillance contributed to the stronger associations in immigrants that was observed when legal registers were included in the analysis. Because the present study cannot distinguish between these potential mechanisms, the findings should be interpreted with caution. In contrast, healthcare utilization and health-related behaviors among immigrants have been shown to gradually converge toward those of the host population with increasing time spent in the receiving country [13, 14, 24]. This may partly explain why the attenuation was less pronounced among second-generation immigrants and why elevated DUD risk remained evident in several of these groups. Overall, the differences in results between the main and sensitivity analyses warrant further investigation into how healthcare utilization and the use of legal registers may influence the identification of substance use disorders among immigrant populations. If lower healthcare utilization contributes to the observed differences, efforts to increase utilization of healthcare services among immigrant populations may facilitate an earlier identification of substance use disorders in this group.

Elevated DUD risk was observed among most immigrant groups and in both males and females. However, the magnitude of the association was generally greater among males. Furthermore, DUD was less common among females than males. The mechanisms behind the lower risks in developing substance use disorders in females may partly be due to social factors. For example, males may have higher access to drugs by having a wider social network outside of the home via the labor market and community [23]. In addition to such differences ascribed to gender roles in societies, cultural and religious norms for substance use in original countries may matter [24]. This is suggested by our supplementary analyses according to immigrants’ regions of origin. These factors might have yielded the findings with a different pattern by sex. However, the gap in substance use between males and females has been narrowing, which may partly be associated with the shift from traditional gender roles to an increased female participation in the society, including the labor market [23]. Therefore, although the absolute occurrence of DUD was lower among females than males, female immigrants should not be overlooked, as several female immigrant groups had higher relative hazards of DUD than non-immigrant females.

Our findings also indicated that both first- and second-generation immigrants were at higher hazards of DUD than non-immigrants. Nonetheless, potential mechanisms for second- and first-generation immigrants to develop DUD may differ. Second-generation immigrants, for example, may be exposed to stress as a result of being born and raised in a different cultural context [25]. First-generation immigrants may, on the other hand, be more affected by adverse experiences before, during, and after their migration. These are important mechanisms that need to be investigated in future studies.

As for AUD, a lower or similar hazard was observed in refugee and non-refugee immigrants compared to non-immigrants. This corroborates previous studies that reported a lower AUD risk among immigrants [12, 26]. Among females, first- and second-generation refugee immigrants had a significantly lower risk of AUD. As mentioned above, this could partly be due to limited access to alcohol among females who come from regions that keep traditional gender roles and where drinking alcohol is restricted by cultural and religious norms [23, 24].

Finally, when region of origin was taken into account, substantial heterogeneity in DUD and AUD risk was observed. In particular, elevated DUD risk was observed among immigrants originating from Africa, Latin America and the Caribbean, and the Middle East or North Africa. These findings suggest that immigrant populations should not be considered a homogeneous group and that migration-related experiences, cultural factors, and social circumstances may contribute differently to substance use disorder risk across populations [8, 9, 24]. In contrast, regional differences in AUD were generally modest, with most immigrant groups showing lower or similar risks compared with non-immigrants. Future studies should further investigate how factors such as pre-migration experiences, social integration, and cultural norms contribute to the observed heterogeneity in substance use disorder risk among immigrant populations.

### Limitations

Our study has several methodological limitations. First, our register-based outcome may not perfectly reflect the rates of substance use disorders. In addition, legal register data can both identify individuals who are not captured in healthcare registers but also include individuals whose legal registrations do not necessarily correspond to a diagnosis. It cannot also be ignored that immigrants might have been more likely to be suspected or convicted for DUD than non-immigrants due to factors such as more often living in neighborhoods with low socioeconomic status where there is greater police monitoring. In addition, the completeness of healthcare and legal register data may have varied over time because of changes in healthcare utilization, diagnostic practices, reporting routines, and registration procedures. Such temporal variation could have influenced the ascertainment of DUD and AUD. To partly account for differences across birth cohorts and calendar periods, birth year was included as a covariate in all adjusted analyses. Second, the presence of unmeasured confounding factors, such as genetic propensity to substance use disorders might have biased our results. Genetic factors are known to account for 50% of the risk of substance use disorder [27]. However, we adjusted for parental substance use disorder from registers, which should have reduced this potential bias. Finally, our study was based on data from Sweden between 2005 and 2020. Since the migration flow is dynamic over time and immigration and integration policies vary between countries, it is important to be aware that our findings may not be applicable to all European countries.

## Conclusions

Our findings showed a higher risk of DUD among certain refugee and non-refugee immigrants than non-immigrants when DUD was measured by healthcare and legal registers. Prevention and treatment strategies for DUD focusing on refugee and non-refugee immigrants should be further examined in healthcare settings supporting refugee and non-refugee immigrants.

## Supplementary Material

ckag152_Supplementary_Data

## Data Availability

The datasets presented in this article are not readily available due to legal concerns. Further information regarding the healthcare register data can be found in the Swedish National Board of Health and Welfare (https://www.socialstyrelsen.se/en/statistics-and-data/registers/). Key Points Refugee and non-refugee immigrants in Sweden, particularly first-generation male refugees, had elevated risk of DUD than non-immigrants. Elevated DUD risk persisted in sensitivity analyses restricted to healthcare data, although the associations were attenuated, particularly among first-generation refugees. Immigrant groups differed substantially according to region of origin, highlighting the heterogeneity of substance use disorder risk among immigrants.
