# Supplementary material for: Substance use disorders among refugee and non-refugee immigrants in Sweden
Source: Eur J Public Health. 2026 Aug 17;36(5):ckag152. doi: 10.1093/eurpub/ckag152 (PMC13479742; doi:10.1093/eurpub/ckag152)
Supplement: Supplementary file 1 [file ckag152_supplementary_data.zip › ejph-2025-12-om-1072-File004.docx]

# **Table S1: Swedish register data used in the study**

| **Data** | **Available year of data and Description** | **Assessed variables** |
| --- | --- | --- |
| Multi-Generation Register | 1932-2020.  Linked children and parents who were born in Sweden or immigrated to Sweden together with parents after 1932.  Country of birth. | Immigrant status. |
| Information on the Reason for Migration | 1997-2020.  Reason for the residence permit in Sweden. | Refugee status. |
| Inpatient Register | 1964-2020.  Hospitalization. | Drug use disorder.  Alcohol use disorder.  Parental substance use disorder^1^. |
| Outpatient Register | 2001-2020.  Hospital visits. | Drug use disorder.  Alcohol use disorder.  Parental substance use disorder^1^. |
| Prescribed Drug Register | 2005-2020.  Prescriptions in Sweden retrieved by patients. | Drug use disorder.  Alcohol use disorder.  Parental substance use disorder^1^. |
| Crime Register | 1973-2020.  Convictions in lower courts of law. | Drug use disorder.  Alcohol use disorder.  Parental substance use disorder^1^. |
| Suspicion Register | 1973-2020.  Suspect of a crime. | Drug use disorder.  Alcohol use disorder.  Parental substance use disorder^1^. |
| Longitudinal Integration Database for Health Insurance and Labor Market Studies (LISA) | 1990-2020.  Socioeconomic information. | Parental education. |
| Cause of Death Register | 1961-2020.  Information of all deaths. | To censor individuals. |

^1^Parental substance use disorder was defined as either drug or alcohol use disorder.
