# Supplementary material for: Substance use disorders among refugee and non-refugee immigrants in Sweden
Source: Eur J Public Health. 2026 Aug 17;36(5):ckag152. doi: 10.1093/eurpub/ckag152 (PMC13479742; doi:10.1093/eurpub/ckag152)
Supplement: Supplementary file 1 [file ckag152_supplementary_data.zip › ejph-2025-12-om-1072-File005.docx]

# **Table S2: Definitions of drug and alcohol use disorders by register and its identification codes that are used in this study**

| **Drug use disorder** | |
| --- | --- |
| Inpatient and Outpatient Register | |
| *ICD-9* | |
| Drug psychoses. | 292 |
| Drug dependence. | 304 |
| Cannabis abuse, Hallucinogens abuse (Include: LSD reaction), Barbiturates and tranquillizers abuse, Morphine type abuse, Cocaine type abuse, Amphetamine type abuse, Antidepressants abuse, Other, mixed or unspecified abuse of drugs. | 305C-305I |
| *ICD-10* | |
| Opioid related disorders, Cannabis related disorders, Sedative, hypnotic, or anxiolytic related disorders, Cocaine related disorders, Other stimulant related disorders, Hallucinogen related disorders, Inhalant related disorders, Other psychoactive substance related disorders. | F11-F16 and F18-F19 |
| Crime and Suspicion Register | |
| *Suspicion and Convictions in lower court* | |
| Driving under the influence of narcotics. | 3070 |
| Use of narcotics. | 5011 |
| Possession^1^ of narcotics. | 5010 |
| Use and possession^1^ of narcotics. | 5012 (available 2000 - 2007) |
| Use and/or possession^1^ of narcotics. | Law 1968:64, paragraph 1, point 6^2^ |
| Driving under the influence of narcotics. | Law 1951:649, paragraph 4, subsection 2 and paragraph 4A, subsection 2 |
| **Alcohol use disorder** | |
| Inpatient and Outpatient Register | |
| *ICD-8* | |
| Alcoholic psychosis, Alcoholism. | 291, 303 |
| Cirrhosis of liver. | 571.0 |
| *ICD-9* | |
| Alcoholic psychoses, Alcohol dependence syndrome, Alcohol Abuse. | 291, 303, 305A |
| Alcohol related complications: Alcoholic polyneuropathy, Alcoholic cardiomyopathy.  Alcoholic gastritis, Alcoholic fatty liver, Alcoholic hepatitis, Alcohol cirrhosis liver, Alcoholic liver damage, unspecified. | 357F, 425F, 535D, 571A, 571B, 571B, 571D |
| *ICD-10* | |
| Mental and behavioral disorders due to use of alcohol, except acute intoxication. | F10.1, F10.2, F10.3, F10.4, F10.5, F10.6, F10.7, F10.8, F10.9 |
| Alcohol related complications: Alcohol-induced pseudo-Cushing syndrome, Alcoholic cardiomyopathy, Alcoholic neuropathy.  Myopathy due to alcohol, Alcoholic cardiomyopathy, Alcoholic cardiomyopathy, Fatty liver due to alcohol, Alcoholic hepatitis, Liver fibrosis and liver sclerosis due to alcohol, Liver cirrhosis due to alcohol, Liver failure due to alcohol.  Unspecified liver injury due to alcohol, Liver failure due to alcohol, Chronic pancreatitis due to alcohol.  Care of pregnant mother with alcohol abuse in which the fetus may be affected. | E24.4, G31.2, G62.1, G72.1, I42.6, K29.2, K70.0, K70.1, K70.2, K70.3, K70.4, K70.9, K85.2, K86.0, O35.4 |
| Prescribed Drug Register | |
| Drugs used in alcohol dependence. | Anatomical Therapeutic Chemical [ATC] Classification System code N07BB; this includes disulfiram (N07BB01), acamprosate (N07BB03), naltrexone (N07BB04) or nalmefene (N07BB05) |
| Crime and Suspicion Register | |
| *Suspicions and Convictions in lower court* | |
| Convicted for, or suspected of at least two alcohol-related crimes, include driving or operating a boat while intoxicated. | Law 1951:649, paragraph 4 and 4A and law 1994:1009, Chapter 20, paragraph 4 and 5 from the Swedish Crime Register, and code 3005 and 3201 in the Suspicion register. |

^1^Possession of narcotics was examined as a potential indicator of drug use disorder. Specifically, over 90% of individuals suspected of possession had at least one other registration for drug use disorders in the Inpatient, Outpatient, Crime, or Suspicion register.

^2^In 2023, the Swedish Penal Law on Narcotics was amended, reducing the number of points from six to five by moving point 6 to point 5.
